# Catechol-O-Methyltransferase Val158Met Polymorphism Associates with Individual Differences in Sleep Physiologic Responses to Chronic Sleep Loss

**DOI:** 10.1371/journal.pone.0029283

**Published:** 2011-12-27

**Authors:** Namni Goel, Siobhan Banks, Ling Lin, Emmanuel Mignot, David F. Dinges

**Affiliations:** 1 Division of Sleep and Chronobiology, Department of Psychiatry, School of Medicine, University of Pennsylvania, Philadelphia, Pennsylvania, United States of America; 2 Center for Narcolepsy, Department of Psychiatry and Behavioral Sciences, Howard Hughes Medical Institute, Stanford University, California, United States of America; Wayne State University, United States of America

## Abstract

**Background:**

The *COMT* Val158Met polymorphism modulates cortical dopaminergic catabolism, and predicts individual differences in prefrontal executive functioning in healthy adults and schizophrenic patients, and associates with EEG differences during sleep loss. We assessed whether the *COMT* Val158Met polymorphism was a novel marker in healthy adults of differential vulnerability to chronic partial sleep deprivation (PSD), a condition distinct from total sleep loss and one experienced by millions on a daily and persistent basis.

**Methodology/Principal Findings:**

20 *Met/Met*, 64 *Val/Met*, and 45 *Val/Val* subjects participated in a protocol of two baseline 10h time in bed (TIB) nights followed by five consecutive 4 h TIB nights. *Met/Met* subjects showed differentially steeper declines in non-REM EEG slow-wave energy (SWE)—the putative homeostatic marker of sleep drive—during PSD, despite comparable baseline SWE declines. *Val/Val* subjects showed differentially smaller increases in slow-wave sleep and smaller reductions in stage 2 sleep during PSD, and had more stage 1 sleep across nights and a shorter baseline REM sleep latency. The genotypes, however, did not differ in performance across various executive function and cognitive tasks and showed comparable increases in subjective and physiological sleepiness in response to chronic sleep loss. *Met/Met* genotypic and *Met* allelic frequencies were higher in whites than African Americans.

**Conclusions/Significance:**

The *COMT* Val158Met polymorphism may be a genetic biomarker for predicting individual differences in sleep physiology—but not in cognitive and executive functioning—resulting from sleep loss in a healthy, racially-diverse adult population of men and women. Beyond healthy sleepers, our results may also provide insight for predicting sleep loss responses in patients with schizophrenia and other psychiatric disorders, since these groups repeatedly experience chronically-curtailed sleep and demonstrate *COMT*-related treatment responses and risk factors for symptom exacerbation.

## Introduction

The *catechol-O-methyltransferase (COMT)* valine158methionine (Val158Met) polymorphism, replaces valine (*Val*) with methionine (*Met*) at codon 158 of the *COMT* protein. As a result of this common substitution, activity of the *COMT* enzyme, which modulates dopaminergic catabolism in the prefrontal cortex (PFC), is reduced 3-to-4-fold in *COMT Met* carriers compared with *Val* carriers, translating into more dopamine availability at the receptors and higher cortical dopamine concentrations [1]. This *COMT* polymorphism functionally predicts less efficient PFC functioning and poor working memory performance on some tasks in healthy subjects [2]–[5] and in patients with schizophrenia [1]–[4], [6] (but see [7]), carrying the high-activity *Val* allele.

In healthy men, the *COMT* Val158Met polymorphism has been associated with sleep physiology. In acute total sleep deprivation (TSD), in which an entire night of sleep is lost, the polymorphism predicted interindividual differences in brain alpha oscillations in wakefulness and 11–13 Hz EEG activity in wakefulness, rapid-eye movement (REM) and non-REM sleep [8]. It also modulated the effects of the wake-promoting drug modafinil on subjective well-being, sustained vigilant attention and executive functioning, and on 3.0–6.75 Hz and >16.75 Hz activity in non-REM sleep, but was not associated with subjective sleepiness, slow-wave activity or slow-wave sleep changes in recovery sleep following TSD or at baseline [9], [10].

In sleep and neurodegenerative disorders, the *COMT* Val158Met polymorphism also has been linked to daytime sleepiness. *Val/Val* female patients with narcolepsy fell asleep two times faster than the *Val/Met* or *Met/Met* genotypes during the Multiple Sleep Latency Test (MSLT) while the opposite was true for males [11]. In addition, *Met/Met* patients with narcolepsy showed more sleep onset REM periods during the MSLT while *Val/Val* subjects showed less sleep paralysis [11] and were more responsive to modafinil's stimulating effects [12]. *Met/Met* and *Val/Met* patients with Parkinson's disease demonstrated higher subjective daytime sleepiness than *Val/Val* subjects [13], although a larger study failed to confirm this finding [14].

Beyond its relationship to sleep and to cognitive function, the *COMT* Val158Met polymorphism has been linked to psychiatric conditions. This polymorphism has been associated with susceptibility to schizophrenia [1], [15] and bipolar disorder [16]–[18] and also has been associated with other mood disorders including major depressive disorder, obsessive compulsive disorder, eating disorders and panic and anxiety disorders [1], [19], [20]. Importantly, this *COMT* polymorphism predicted behaviors in healthy adults which tie to psychiatric disorders [2], [3]—thus, investigation of this genotype in healthy individuals has applications for clinical research.

Considering the aforementioned literature, we hypothesized that *COMT* would be a novel genetic biomarker in healthy adult sleepers of differential vulnerability to sleep homeostatic, sleepiness and neurobehavioral responses during chronic PSD. Chronic PSD is a condition representative of real world situations, experienced by millions on a consecutive and persistent basis, and associated with serious health consequences [21]. In addition, chronic PSD is similar to the sleep-wake patterns found in schizophrenia—and in bipolar disorder and other mood disorders—whereby patients experience repeatedly curtailed or fragmented sleep rather than lose an entire night of sleep [22]. Thus, as an ancillary objective, we investigated responses to such PSD conditions in healthy adults as a putative experimental model for predicting sleep, alertness and cognitive responses to sleep loss in patients with psychiatric disorders.

## Materials and Methods

### Ethics Statement

The protocols described below were approved by the Institutional Review Board of the University of Pennsylvania. After complete description of the study, and prior to study entry, written informed consent was obtained from all subjects according to the principles expressed in the Declaration of Helsinki; all subjects received compensation for participation.

### Participants

One hundred and twenty-nine subjects participated in one of two chronic PSD experiments (described below). Following protocol completion, subjects were genotyped for the *COMT* Val158Met polymorphism. This was a retrospective analysis; thus, *COMT* genotypes were not matched. The *COMT* Val158Met genotypic and allelic frequencies for whites and African Americans (Table 1) approximated those reported in other studies using mixed ethnicity samples [23].

**Table 1:** Characteristics of *Met/Met, Val/Met* and *Val/Val* Subjects (Mean ± SD).

Characteristic	*Met/Met*	*Val/Met*	*Val/Val*	p*
**N (%)**	20 (15.5%)	64 (49.6%)	45 (34.9%)	
**Age**	29.7±6.9	29.9±6.7	30.2±7.3	0.969
**BMI (kg/m^2^)**	24.98±3.91	24.91±3.05	24.19±3.90	0.248
**Sex (M/F)**	7/13	35/29	24/21	0.277
**Ethnicity (White/African American/Other)** #	14(.29)/6(.08)/0(.00)	23(.47)/39(.51)/2(.50)	12(.24)/31(.41)/2(.50)	
**Morningness-Eveningness Composite Scale**	39.35±5.91	39.86±6.06	40.19±5.04^a^	0.978
**Epworth Sleepiness Scale**	4.45±2.86	4.95±3.08	4.86±3.16^b^	0.902
**Beck Depression Inventory (BDI)**	1.47±2.29^c^	1.27±1.77^d^	1.82±2.77	0.485
**Eysenck Personality Inventory (Extraversion subscale)**	15.11±4.45^c^	15.08±3.93^e^	15.88±3.44^a^	0.377
**North American Adult Reading Test (IQ)**	109.08±8.34	107.14±7.13^f^	102.79±8.45† ^g^	0.028
**Sleep Onset by Actigraphy** **	23∶50±1.20 h	24∶00±0.88 h^d^	23∶44±0.80 h^b^	0.363
**Sleep Offset by Actigraphy** **	08∶02±1.27 h	07∶54±0.92 h^d^	07∶46±0.85 h^b^	0.748
**Sleep Midpoint by Actigraphy** **	04∶06±0.33 h	03∶57±0.32 h^d^	04∶01±0.37 h^b^	0.448
**Total Sleep Time** ** **(Sleep Duration)**	8.21±0.70 h	7.94±0.70 h^d^	8.03±0.74 h^b^	0.592

a n = 42.

b n = 44.

c n = 19.

d n = 63.

e n = 62.

f n = 61.

g n = 43.

† Lower than *Met/Met* and *Val/Met*, p<0.05, Bonferroni correction.

*p values are for the comparison of the three genotypes.

**One week prior to study entry.

# Genotypic frequencies are in parentheses. *Met* allele frequency was .520 for whites and .340 African Americans; *Val* allele frequency was .480 for whites and .660 African Americans. The ethnicities showed *Met/Met* genotypic (χ^2^ = 9.48, p = 0.002) and allelic differences (χ^2^ = 8.43, p = 0.004).

Subjects met the following inclusionary criteria, as detailed in [24]: age between 22–45 yrs.; physically and psychologically healthy, as assessed by physical examination and history; no clinically significant blood chemistry abnormalities; drug-free urine samples; good habitual sleep, between 6.5–8.5 h daily duration with regular bedtimes, and wake-up times between 0600–0900 h (verified by sleep logs and wrist actigraphy for ≥one week before study entry); absence of extreme morningness/eveningness; absence of sleep or circadian disorders, assessed by questionnaire and polysomnography; absence of psychiatric illness or adverse neuropsychiatric reaction to sleep deprivation; no alcohol or drug abuse history; no current use of medical or drug treatments (excluding oral contraceptives).

### Experimental Design

Subjects participated in an 11- or 16-day experiment in the Sleep and Chronobiology Laboratory at the Hospital of the University of Pennsylvania. Only data from the first seven nights of the protocols—which were procedurally identical—were analyzed. On the two baseline nights, subjects received 10 h time in bed (TIB) from 2200–0800 h to reduce any pre-existing sleep debt; on the subsequent five nights, subjects received 4 h TIB (0400–0800 h).

During the protocol, laboratory conditions and scheduled activities were highly controlled. Ambient light remained at <50 lux during wakefulness, and at <1 lux (darkness) during sleep. Temperature was maintained at 22±1°C. Subjects were continuously monitored by trained staff. Between performance bouts, they were restricted from strenuous activities or having visitors, but could read, play games, watch movies, and interact with staff to help remain awake. Subjects received three standardized meals per day and an optional evening snack. Caffeine, turkey, bananas, alcohol and tobacco were prohibited.

### Neurobehavioral Assessments

Subjects performed a computerized neurobehavioral test battery every 2 h during wakefulness, as detailed in [24], which included: the Karolinska Sleepiness Scale (KSS), a Likert-type subjective sleepiness scale; a visual analog scale of fatigue (VAS) anchored by “fresh as a daisy” and “tired to death”; the Profile of Mood States (POMS), a scale assessing transient affective states; the Digit Symbol Substitution Task (DSST), a cognitive throughput task; the Digit Span (DS) task, a working memory storage capacity test, given in forward and backward versions and summed as a total number correct measure; and the Psychomotor Vigilance Task (PVT), a sustained attention test utilizing reaction times as a behavioral alertness assay. Subjects remained seated throughout testing, were behaviorally monitored, and were instructed to perform to the best of their ability and use compensatory effort to maintain performance. Baseline values were derived from the second baseline day (B2). Daily values for each performance task were calculated by averaging scores from all test bouts that day.

### Other Measurements

Before the study, subjects completed questionnaires on demographic, clinical sleepiness, sleep–wake and circadian-related variables, and psychosocial/personality traits, as detailed in [24], including the Epworth Sleepiness Scale, the Morningness-Eveningness Composite Scale, the Beck Depression Inventory, the Eysenck Personality Inventory and the North American Adult Reading Test. At partial sleep deprivation/restriction night 5 (SR5), four standardized executive function tests were administered: the Hayling and Brixton tests, the Controlled Oral Word Association Test, and the Tower of London. In the 11-day protocol, a modified Maintenance of Wakefulness Test (MWT) [25]—a physiological measure of the ability to resist sleep—was administered at B2 and SR5 (a single trial was conducted between 1430–1600 h) using a standard recording montage. Before each trial, the lights were dimmed to <10 lux and subjects were instructed to “keep your eyes open and try not to fall asleep”. Each trial was terminated at the first microsleep (10 seconds of theta activity) [25] determined by the C3/A2 derivation or at 30 minutes if sleep onset did not occur. MWT scores represented either the time (minutes) to microsleep initiation or 30 minutes (if no microsleep occurred).

### Sleep Architecture

#### Polysomnography

The polysomnographic (PSG) montage included frontal (Fz), central (C3), and occipital (O2) EEG, bilateral EOG, submental EMG, and ECG. Data were recorded from 2200–0800 h on B2, and from 0400–0800 h on partial sleep deprivation/restriction night 1 (SR1) and SR5. Records were visually scored in 30-second epochs using standard scoring criteria by a trained scorer blind to *COMT* typing.

#### EEG Analysis

**Figure 1:**
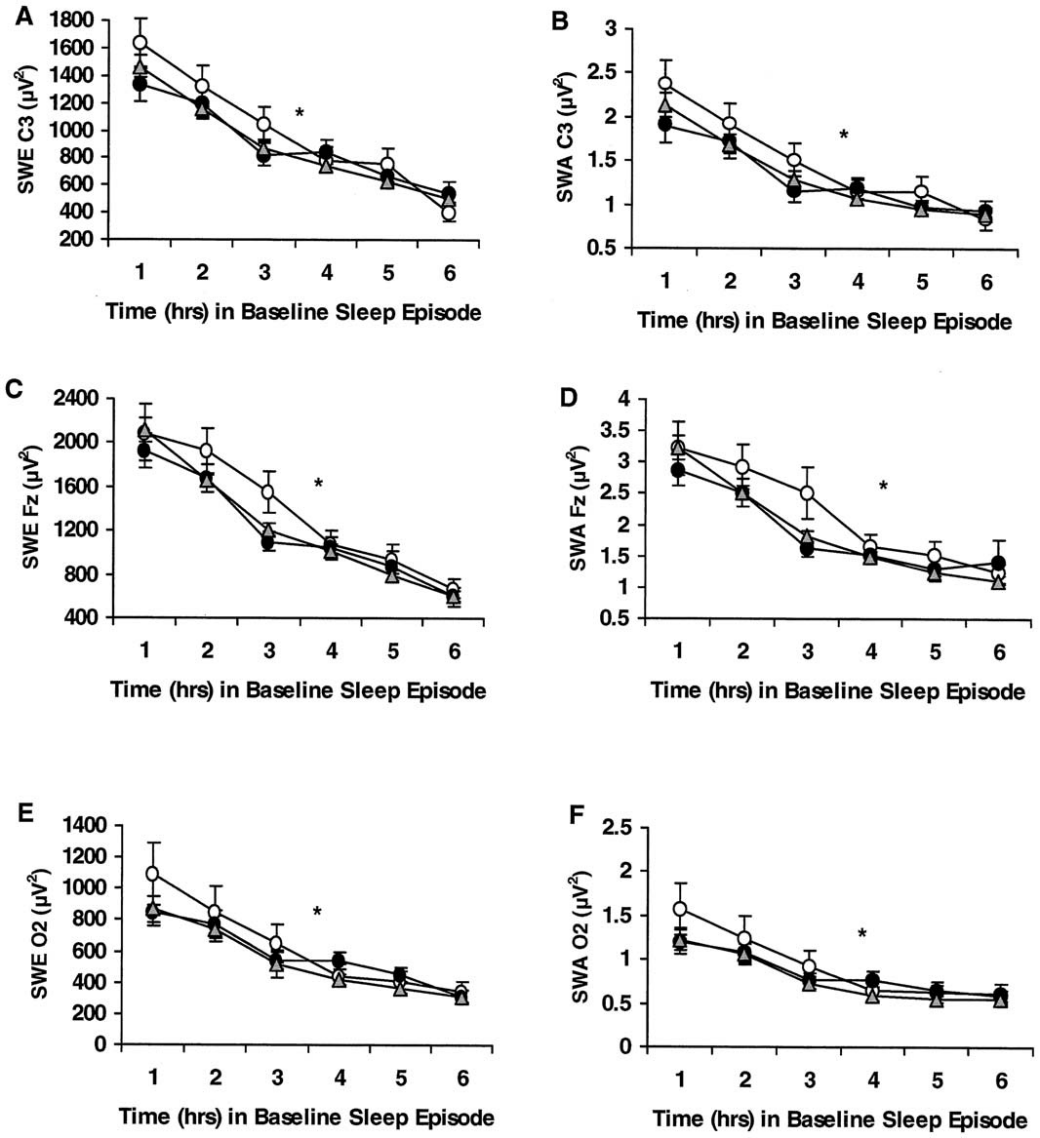
Hourly slow-wave energy (SWE) and slow-wave activity (SWA) during baseline for the *COMT* Val158Met genotypes. Mean (±SEM) hourly SWE and SWA derived from the C3 (A, B), Fz (C, D) or O2 (E, F) channels during baseline for *Met/Met (open circles)*, *Val/Met (gray triangles)* and *Val/Val (closed circles)* subjects. As expected, SWE and SWA showed a typical pattern of dissipation across the baseline night in all 3 channels in all genotypes (denoted by *, p<0.0001), but did not show a differential pattern of decline across genotypes. The groups also did not show significant differences in SWE or SWA derived from the C3, Fz or O2 channels. In some records, EEG signal quality was insufficient or contained too much artifact for reliable power spectral analysis. Thus, the final sample sizes were as follows: for C3, *Met/Met* (n = 19), *Val/Met* (n = 60), and *Val/Val* (n = 39) subjects; for Fz, *Met/Met* (n = 18), *Val/Met* (n = 62), and *Val/Val* (n = 42) subjects; for O2, *Met/Met* (n = 19), *Val/Met* (n = 61), and *Val/Val* (n = 44) subjects.

**Figure 2:**
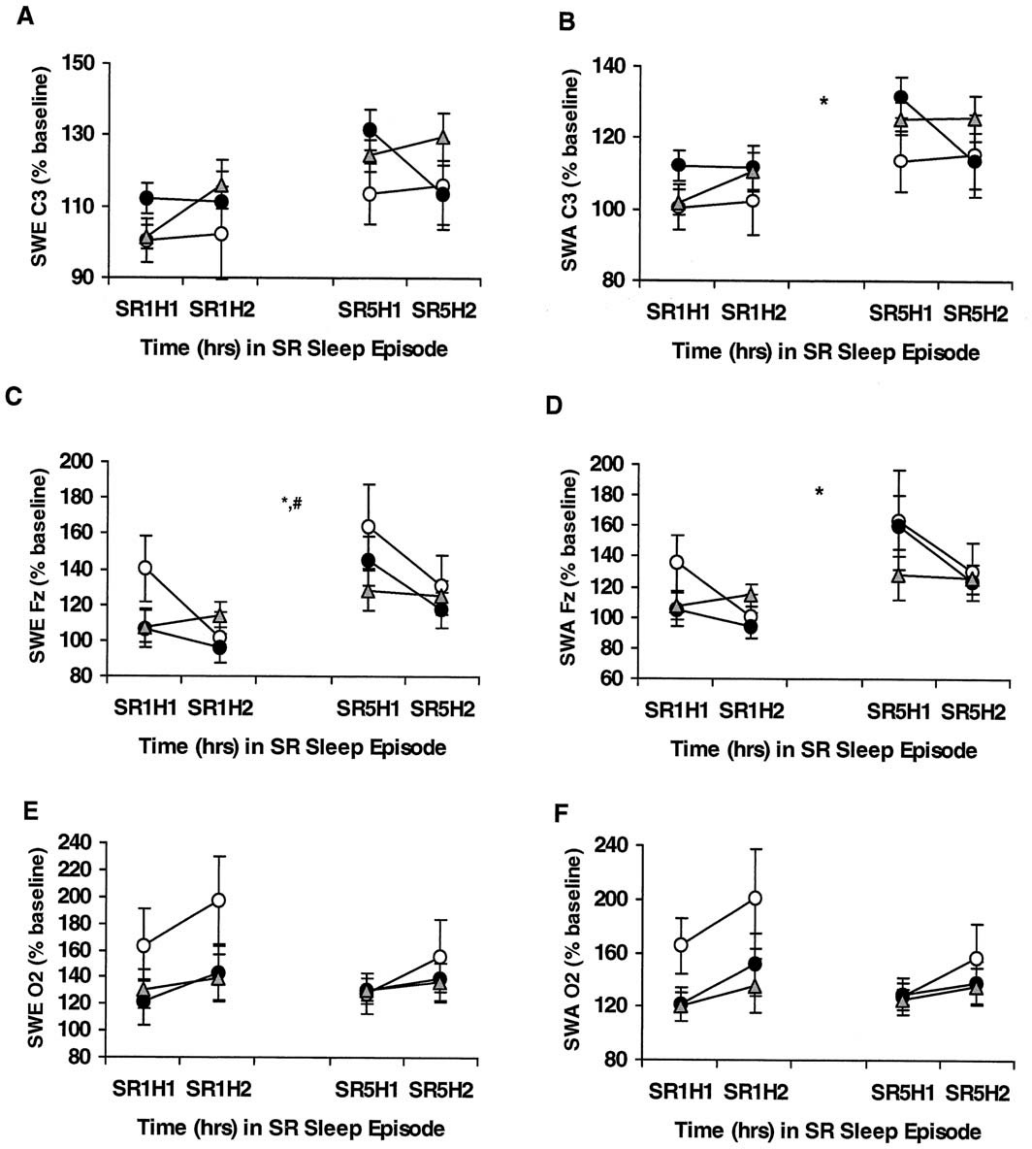
Slow-wave activity (SWA) and slow-wave energy (SWE) during sleep deprivation for the *COMT* Val158Met genotypes. Mean (±SEM) hourly SWA and SWE as a percentage of baseline at the same corresponding hour derived from the C3 (A, B), Fz (C, D) or O2 (E, F) channels on partial sleep deprivation/restriction night 1 (SR1) and partial sleep deprivation/restriction night 5 (SR5) for hour 1 (H1) and hour 2 (H2) in *Met/Met (open circles)*, *Val/Met (gray triangles)* and *Val/Val (closed circles)* subjects. *Met/Met* subjects showed differentially greater dissipation during sleep restriction nights in NREM EEG SWE (derived from the Fz channel)—the putative homeostatic marker of sleep drive—compared with *Val/Met and Val/Val* subjects (denoted by ^#^, p<0.05). SWA and SWE derived from the Fz and C3 channels increased from SR1 to SR5 (denoted by *, p<0.05). In some records, EEG signal quality was insufficient or contained too much artifact for reliable power spectral analysis. Thus, the final sample sizes were as follows: for SR1 and SR5 C3, *Met/Met* (n = 15), *Val/Met* (n = 56) and *Val/Val* (n = 37) subjects; for SR1 and SR5 Fz, *Met/Met* (n = 13), *Val/Met* (n = 54) and *Val/Val* (n = 36) subjects; for SR1 and SR5 O2, *Met/Met* (n = 14), *Val/Met* (n = 48) and *Val/Val* (n = 36) subjects.

After visually determined artifact rejection, the EEG was sampled at 128 Hz and spectral analysis of 3 sleep EEG derivations (C3/A2; Fz/A1; O2/A1) was performed with Fast Fourier Transform averaged across consecutive 30-second epochs (average of 6 5-second epochs, resulting in a frequency resolution of 0.2 Hz). We chose to examine the C3, Fz and O2 derivations, since we have found differential genotype changes in these derivations in prior studies [24]. For each night, slow-wave energy (SWE) in the delta band (0.5–4.5 Hz) was totaled over all epochs of non-REM (visually-scored stages 2–4) sleep. Power in the delta band (SWA) was calculated by dividing SWE by the number of non-REM sleep epochs. For B2, absolute values were determined for each hour of sleep for SWE and SWA; for SR1 and SR5, SWE and SWA were normalized by calculating the percent of the corresponding B2 hour. For some records, EEG signal quality was insufficient or contained too much artifact for reliable power spectral analysis (Figures 1 and 2). Although the delta band was of primary interest, we also examined other frequency bands in both non-REM and REM sleep which were defined as follows: theta (4.5–8 Hz), alpha (8–12 Hz), sigma (12–14 Hz), and beta (14–30 Hz).

### Genotyping

Genomic DNA was extracted from whole blood using Qiagen's QIAamp DNA Blood Mini Kit (Catalog #51106). *COMT* genotypes were determined by PCR-RFLP analysis. A G-to-A substitution at codon 158 encoding valine or methionine generates this polymorphism. The target sequence was PCR-amplified and the product was digested by the restriction enzyme Nla III and electrophoresed in an 8% polyacrylamide gel to detect the two alleles, as described in [26].

### Statistical Analyses

Mixed model analyses of covariance (ANCOVA), with day or hour as the within-subjects (repeated measures) factor, genotype as the between-group factor, and ethnicity as a covariate, were used to analyze MWT, PSG, EEG, PVT, KSS, VAS, POMS, DSST and DS data. Greenhouse–Geisser corrections were applied to all within-subjects effects. One-way ANCOVA, with ethnicity as a covariate, were used to analyze demographic and pre-study measures, PSG, MWT and cognitive, executive function and sleep measures during baseline and chronic PSD. In separate analyses, gender was investigated as an additional factor because of previous findings [11]; however, no significant gender differences were found. Post-hoc comparisons using Bonferroni-adjusted probabilities examined significant group differences for all measures. SPSS version 15.0 (SPSS Inc., Chicago, IL) was used for statistical analyses; p≤0.05 was considered significant.

## Results

### Demographic and Pre-study Variables

There were *Met/Met* genotypic differences (χ^2^ = 9.48, p = 0.002) and *Met* allelic differences (χ^2^ = 8.43, p = 0.004) between whites and African Americans (Table 1); therefore, ethnicity was statistically controlled for as a covariate in all analyses. *Met/Met* and *Val/Met* individuals had higher IQ scores than *Val/Val* subjects (Table 1), although the groups did not differ significantly in other demographic variables including age, body mass index, or sex. Moreover, the groups did not show differences in psychosocial/personality traits, pre-study sleep variables, clinical sleepiness or circadian phase markers (Morningness-Eveningness chronotype and sleep midpoint).

### Sleep Physiology

#### Non-REM EEG Slow-Wave Energy and Slow-Wave Activity

Across B2, the genotypes did not differ in SWE or SWA calculated from the C3 (Figure 1A, 1B; SWE: *F*_2,88_ = 1.12, p* = *0.332; SWA: *F*_2,88_ = 0.55, p* = *0.577), Fz (Figure 1C, 1D; SWE: *F*_2,94_ = 0.22, p* = *0.801; SWA: *F*_2,94_ = 0.31, p* = *0.737) and O2 (Figure 1E, 1F; SWE: *F*_2,97_ = 0.61, p* = *0.545; SWA: *F*_2,97_ = 0.58, p* = *0.561) EEG derivations. SWE and SWA dissipated across B2 for the C3 (SWE: *F*_3.36,295.63_ = 38.40, p*<*0.0001; SWA: *F*_3.02,265.85_ = 36.51, p*<*0.0001), Fz (SWE: *F*_2.41,226.56_ = 18.16, p*<*0.0001; SWA: *F*_1.92,180.49_ = 11.63, p*<*0.0001) and O2 channels (SWE: *F*_1.97,190.58_ = 15.13, p*<*0.0001; SWA: *F*_1.85,179.55_ = 13.19, p*<*0.0001), but not in a differential pattern across genotypes (C3 SWE: *F*_6.72, 295.63_ = 0.93, p* = *0.480; C3 SWA: *F*_6.04,265.85_ = 0.76, p* = *0.603; Fz SWE: *F*_4.82,226.56_ = 0.57, p* = *0.720; Fz SWA: *F*_3.84,180.49_ = 0.76, p* = *0.550; O2 SWE: *F*_3.93,190.58_ = 0.47, p* = *0.754; O2 SWA: *F*_3.70,179.55_ = 0.45, p* = *0.758).

SWE and SWA displayed acute responses to PSD in all groups—evidenced by percentage increases above the corresponding B2 hour in all channels. SWE% baseline showed a differential change across PSD for the *COMT* genotypes in the Fz derivation (Figure 2C; *F*_4.55,213.81_ = 2.57, p* = *0.032), but not in Fz SWA% baseline (Figure 2D; *F*_3.48,165.39_ = 2.11, p* = *0.092) with *Met/Met* subjects showing sharper dissipation of SWE from hour 1 to hour 2 on SR1 and SR5. By contrast, neither SWA% baseline nor SWE% baseline in the C3 (Figure 2A, 2B; SWA% baseline: *F*_4.19,201.25_ = 0.86, p* = *0.490; SWE% baseline: *F*_3.76,180.25_ = 1.25, p* = *0.294) or O2 EEG derivations showed significant differential changes across chronic PSD (Figure 2E, 2F; SWA% baseline: *F*_4.25,197.47_ = 0.72, p* = *0.589; SWE% baseline: *F*_4.52,212.28_ = 0.75, p* = *0.574).

SWA% baseline and SWE% baseline derived from Fz increased across PSD (Figure 2C, 2D; SWA% baseline: *F*_1.74,165.39_ = 3.93, p* = *0.027; SWE% baseline: *F*_2.28,213.81_ = 4.08, p* = *0.014), as did SWA% baseline derived from C3 (Figure 2B; *F*_2.10,201.25_ = 3.13, p* = *0.044), while no other measures were significant (Figure 2A, 2E, 2F; SWE C3% baseline: *F*_1.88,180.25_ = 1.80, p* = *0.171; SWE O2% baseline: *F*_2.26,212.28_ = 2.32, p* = *0.094; SWA O2% baseline: *F*_2.12,197.47_ = 1.59, p* = *0.205). In addition, the groups did not differ in SWE% baseline (*F*_2,96_ = 0.78, p* = *0.460) or SWA% baseline (*F*_2,96_ = 1.20, p* = *0.307) from the C3 (Figure 2A, 2B), Fz (Figure 2C, 2D; SWE% baseline: *F*_2,94_ = 0.32, p* = *0.727; SWA% baseline: *F*_2,95_ = 0.10, p* = *0.901) or O2 channels (Figure 2E, 2F; SWE% baseline: *F*_2,94_ = 0.84, p* = *0.436; SWA% baseline: *F*_2,93_ = 1.41, p* = *0.248).

#### Other EEG Frequencies

Although the primary focus of our EEG analyses was on the delta frequency in NREM, as the putative marker of sleep homeostasis, we also examined the other EEG frequency ranges in NREM and in REM sleep at baseline and during sleep restriction for the C3, Fz, and O2 derivations. We failed to find significant genotype differences for any of these measures (all p's>0.05).

#### Polysomnography

Across B2, SR1 and SR5, *Val/Val* subjects had more stage 1 sleep (Table 2; duration: *F*_2,99_ = 3.15, p = 0.047; %TST: *F*_2,99_ = 3.72, p = 0.028, Bonferroni correction, p<0.05). At B2, these subjects had shorter REM sleep latency (*F*_2,113_ = 3.07, p = 0.050, Bonferroni correction, p<0.05) and more stage 1 sleep (duration: *F*_2,113_ = 3.95, p = 0.022; %TST: *F*_2,113_ = 3.30, p = 0.040, Bonferroni correction, p<0.05). This latter difference was maintained at SR1, with *Val/Val* subjects showing more stage 1 sleep (duration: *F*_2,113_ = 5.50, p = 0.005; %TST: *F*_2,113_ = 6.16, p = 0.003, Bonferroni correction, p<0.05).

**Table 2:** Polysomnographic Sleep Measures during B2, SR1 and SR5 for *Met/Met*, *Val/Met* and *Val/Val* Subjects (Mean ± SD)

Sleep Measure.	Baseline Night (B2)				Sleep Restriction Night 1 (SR1)				Sleep Restriction Night 5 (SR5)			
	*Met/Met*	*Val/Met*	*Val/Val*	p*	*Met/Met*	*Val/Met*	*Val/Val*	p*	*Met/Met*	*Val/Met*	*Val/Val*	p*
**Total sleep time (min)**	495.79±61.82	515.35±59.31	522.22±45.49	0.157	225.66±8.74	226.84±9.45	224.18±8.57	0.377	232.35±5.95	232.08±7.28	232.95±7.42	0.669
**Sleep efficiency (%)**	82.64±10.29	86.42±9.75	87.58±7.71	0.126	94.19±3.80	94.66±3.90	93.52±3.45	0.354	96.64±2.50	96.66±3.06	97.09±3.09	0.519
**Sleep onset latency (min)**	25.87±24.99	17.80±20.85	14.54±14.81	0.233	3.28±3.91	3.19±4.30	4.06±5.13	0.582	2.98±3.73	2.14±3.74	1.57±2.67	0.124
**REM sleep latency (min)**	93.00±39.53	74.52±31.11	67.03±38.92†	0.050	52.34±31.93	56.80±29.67	49.77±27.61	0.469	41.73±25.50	43.73±30.07	39.83±22.36	0.507
**WASO (min)**	71.63±61.01	57.50±55.79	48.59±40.45	0.165	8.00±8.42	5.62±7.55	7.13±8.06	0.335	3.60±3.48	3.62±5.50	2.33±2.56	0.128
**Awakenings (number)**	16.16±11.94	16.54±12.16	13.28±10.11	0.203	4.50±3.25	3.60±3.18	4.00±3.92	0.456	3.15±2.87	3.03±3.24	2.19±1.83	0.077
**Stage 1 duration (min)** a	43.34±25.07	51.29±26.06	62.15±28.33†	0.022	14.03±8.75	12.36±8.14	18.68±10.82††	0.005	8.68±4.31	7.78±5.44	10.53±7.07	0.095
**Stage 1 (%TST)** a	8.69±4.67	10.03±4.94	12.02±5.61	0.040	6.31±4.14	5.46±3.57	8.48±4.94††	0.003	3.77±1.90	3.36±2.37	4.57±3.14	0.100
**Stage 2 duration (min)**	263.53±63.99	279.96±47.44	274.18±50.26	0.647	98.81±40.04	102.80±29.72	96.26±22.90	0.271	89.45±34.25	96.99±27.95	105.58±32.56	0.518
**Stage 2 (%TST)** b	52.92±9.89	54.56±8.24	52.46±7.89	0.188	43.98±18.22	45.40±13.19	43.04±10.35	0.353	38.63±15.02	41.92±12.43	45.34±13.86	0.606
**Stage 3 duration (min)**	39.08±19.90	41.85±18.55	40.59±17.22	0.540	24.44±13.39	32.91±12.56	31.95±14.63	0.074	27.23±14.67	35.21±14.48	32.30±15.28	0.185
**Stage 3 (%TST)**	7.93±4.03	8.20±3.68	7.77±3.34	0.770	10.82±6.03	14.51±5.51	14.20±6.35	0.076	11.71±6.32	15.15±6.22	13.83±6.52	0.179
**Stage 4 duration (min)**	33.29±26.58	24.95±25.62	25.18±27.91	0.868	34.91±27.08	30.01±27.41	26.29±26.10	0.888	40.40±26.10	36.58±28.88	26.59±30.07	0.407
**Stage 4 (%TST)**	7.02±5.96	4.69±4.82	4.75±5.19	0.676	15.38±11.81	13.16±11.89	11.69±11.50	0.912	17.36±11.21	15.66±12.26	11.34±12.80	0.392
**SWS duration (min)**	72.37±36.90	66.80±36.93	65.77±38.80	0.850	59.34±33.84	62.92±28.10	58.24±29.83	0.526	67.63±26.79	71.78±28.71	58.90±33.88	0.139
**SWS (%TST)** c	14.94±7.93	12.90±6.84	12.53±7.30	0.971	26.20±14.89	27.67±12.10	25.89±13.14	0.559	29.07±11.45	30.81±12.10	25.17±14.39	0.127
**REM sleep duration (min)**	116.53±26.13	117.26±31.87	120.09±27.46	0.804	53.47±21.38	48.72±14.86	50.96±14.81	0.616	63.60±13.33	55.58±16.79	57.85±16.14	0.289
**REM sleep (%TST)**	23.45±4.16	22.50±4.85	22.99±4.75	0.749	23.51±8.99	21.43±6.25	22.72±6.58	0.503	27.40±5.54	23.80±7.01	24.88±7.08	0.229

*p values are for the comparison of the three genotypes. Equipment problems resulted in some data loss. Final sample sizes were: at B2, *Met/Met* (n = 19), *Val/Met* (n = 59), and *Val/Val* (n = 39); at SR1, *Met/Met* (n = 16), *Val/Met* (n = 60), and *Val/Val* (n = 41); at SR5, *Met/Met* (n = 20), *Val/Met* (n = 58), and *Val/Val* (n = 43).

† Different from *Met/Met*, p<0.05, Bonferroni correction.

†† Different from *Val/Met*, p<0.05, Bonferroni correction.

a Genotype effects (Stage 1 duration: *F*_2,99_ = 3.15, p = 0.047; Stage 1 %TST: *F*_2,99_ = 3.72, p = 0.028).

b Night × genotype interaction (F_3.87,191.578_ = 2.65, p = 0.036).

c Night × genotype interaction (F_3.92,194.083_ = 2.81, p = 0.027).

Abbreviations: Total Sleep Time (TST); Wake After Sleep Onset (WASO); Slow-Wave Sleep (SWS); Rapid Eye Movement (REM).

The genotypes showed differential PSG responses to PSD (Table 2). During PSD, *Val/Val* subjects showed differentially smaller increases in SWS (%TST: F_3.92,194.083_ = 2.81, p = 0.027) and smaller reductions in stage 2 sleep (%TST: F_3.87,191.578_ = 2.65, p = 0.036). All genotypes displayed acute responses consistent with sleep loss and with increases in homeostatic drive: sleep efficiency and stages 3 and 4 (slow-wave) sleep significantly increased, while TST, sleep onset latency, WASO, and stages 1 and 2 sleep significantly decreased (Table 2).

### Cognitive Performance and Executive Functioning

**Figure 3:**
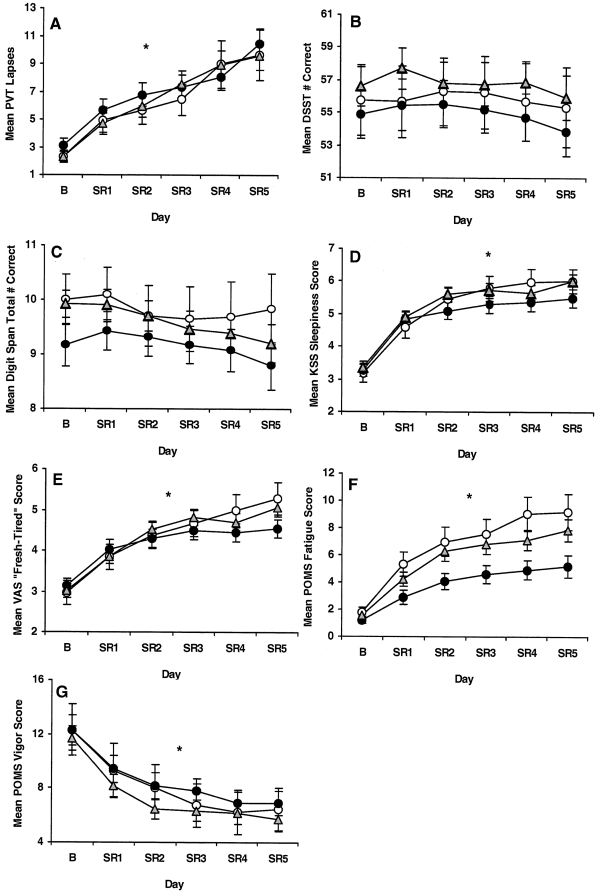
Neurobehavioral performance during baseline and chronic partial sleep deprivation for the *COMT* Val158Met groups. Mean (±SEM) (A) PVT lapses (>500 ms reaction times) per trial, (B) total number correct per trial on the Digit Symbol Substitution Task (DSST) and on the (C) Digit Span (DS) task, and scores per trial on the (D) Karolinska Sleepiness (KSS), (E) “Fresh-Tired” Visual Analog Scale (VAS), (F) POMS-Fatigue scale and (G) POMS-vigor scale at baseline (B) and each partial sleep deprivation/restriction night (SR1-SR5) for *Met/Met (open circles)*, *Val/Met (gray triangles)* and *Val/Val (closed circles)* subjects. Although all genotypes showed increased PVT lapses (denoted by *, p<0.0001) and variability across chronic PSD, there were no differential responses in lapses nor did one genotype show more lapses than the other groups at baseline or during chronic PSD. There were no group differences or differential changes in DSST or DS scores across chronic PSD nor were there significant changes across days. For all genotypes, POMS-Vigor scores decreased, and KSS, VAS and POMS-Fatigue scores increased across chronic PSD (denoted by *, p<0.0001), but there were no differential changes or group differences in these measures during chronic PSD.

Chronic PSD induced significant cognitive performance deficits across days as demonstrated by increases in PVT lapses (>500ms reaction times) and in variability for all groups across sleep loss (Figure 3A). Although all genotypes increased lapses across days (*F*_2.45,305.67_ = 12.90, p*<*0.0001), there were no differential responses (*F*_4.89,305.67_ = 0.92, p = 0.469) or group differences across days (*F*_2,125_ = 0.06, p = 0.941). Moreover, lapses and other PVT measures—errors, fastest 10% and median reaction times, response speed—did not differ across groups during B2 or PSD (all p's>0.05).

The groups showed no differential changes in DSST performance across PSD (*F*_5.77,360.44_ = 0.50, p* = *0.802) nor did scores change across days (*F*_2.88,360.44_ = 1.96, p* = *0.123) or differ across groups across days (Figure 3B; *F*_2,125_ = 0.66, p* = *0.521). There were no B2 or PSD group differences in DSST performance (all p's>0.05). Similarly, there were no differential changes in DS total performance across PSD (*F*_5.54,346.18_ = 0.67, p* = *0.660) or group differences across days (*F*_2,125_ = 0.35, p* = *0.707) nor did scores change across days (Figure 3C; *F*_2.77,346.18_ = 0.88, p* = *0.444). Moreover, the groups did not show DS performance differences during B2 or PSD (all p's>0.05).

The Hayling, Brixton, Controlled Oral Word Association Test, and Tower of London—executive function tests measured at SR5—showed no significant differences across the *COMT* genotypes (all p's>0.05).

### Subjective Sleepiness and Fatigue

PSD produced increases in KSS sleepiness (Figure 3D; *F*_3.00,374.85_ = 17.94, p*<*0.0001) and VAS fatigue scores (Figure 3E; *F*_2.48,309.02_ = 20.96, p*<*0.0001) for all genotypes across days. Despite such increased scores across chronic PSD, there were no differential responses in these measures (KSS: *F*_6.00,374.85_ = 1.28, p* = *0.268; VAS: *F*_4.94,309.02_ = 1.68, p* = *0.140) or group differences across days (KSS: *F*_2,125_ = 0.49, p* = *0.615; VAS:*F*_2,125_ = 0.15, p* = *0.858). Similarly, on an unrelated scale, the Profile of Mood States (POMS), PSD produced increases in subjective fatigue (POMS-F; Figure 3F; *F*_2.34,292.27_ = 38.01, p*<*0.0001) and decreases in vigor (POMS-V; Figure 3G; *F*_2.35,293.41_ = 8.79, p*<*0.0001) across days for all genotypes, but no differential responses (POMS-F: *F*_4.68,292.27_ = 1.43, p* = *0.217; POMS-V: *F*_4.70,293.41_ = 0.66, p* = *0.643) or group differences across days for either measure (POMS-F: *F*_2,125_ = 2.59, p* = *0.079; POMS-V: *F*_2,125_ = 0.46, p* = *0.632). Moreover, the genotypes did not differ on KSS, VAS, POMS-F or POMS-V scores during B2 or PSD (all p's>0.05).

### Physiological Sleepiness

Substantiating the subjective sleepiness data, MWT scores did not differ across groups (*F*_2,86_ = 0.12, p* = *0.889) or show differential changes to PSD (*F*_2,86_ = 0.05, p* = *0.951), although all genotypes were less able to resist sleep following deprivation (*F*_1,86_ = 4.88, p = 0.030). The groups did not differ on MWT scores at B2 (*F*_2,86_ = 0.14, p* = *0.936) or SR5 (*F*_2,86_ = 0.02, p* = *0.882).

## Discussion

The *COMT* Val158Met polymorphism related to individual differences in sleep homeostatic responses and physiological sleep responses to chronic PSD. *Met/Met* subjects showed differentially greater declines across days of PSD in NREM EEG SWE—the putative homeostatic marker of sleep drive—compared with *Val/Met and Val/Val* subjects, despite comparable baseline declines. *Val/Val* subjects showed differentially smaller SWS increases and smaller reductions in stage 2 sleep during PSD, had more stage 1 sleep across nights, and a shorter baseline REM sleep latency—all indicative of a lower homeostatic drive. The genotypes demonstrated comparable cumulative decreases in cognitive performance, and increases in subjective and physiological sleepiness and fatigue to PSD, with increasing daily inter-subject variability, and showed no executive function performance differences. The *COMT* Val158Met polymorphism may be a genetic marker for predicting individual differences in sleep homeostasis and physiology, but not in cognitive and executive function responses, resulting from sleep loss in a healthy, racially-diverse population of men and women. Furthermore, these genotype differences in SWE in response to PSD may extend to psychiatric populations; they may relate to treatment response in depression and schizophrenia and may protect against development and exacerbation of psychosis in these disorders.

Under the phenotypic conditions elicited by PSD, *Met/Met* subjects had significantly larger declines in SWE. Moreover, compared with *Val/Val* subjects, *Met/Met* subjects showed significantly less stage 1 sleep and a longer REM sleep latency at baseline and chronic PSD—indicative of a greater sleep homeostatic drive. Since such differences were not observed under basal, fully-rested homeostatic pressure conditions, as was similarly reported in another study [9], *Met/Met* subjects may possess a greater drive, coupled with a more efficient homeostatic response to sleep loss. Other frequencies, including alpha, which in prior work has shown *COMT* genotype differences [8], did not show genotype-dependent differences in our study. This finding suggests that the *COMT* Val158Met polymorphism's influence is likely specific to SWA/SWE and is not due to nonspecific effects of the *COMT* genotype on EEG-generating mechanisms. Different genes may modulate basal versus evoked homeostatic responses in healthy sleepers; therefore, other markers may influence differential vulnerability in fully-rested conditions.

The *COMT* polymorphism related to individual differences in sleep homeostatic and physiological responses to chronic PSD, contrasting observations in acute TSD [9]. This difference may be due to the nature of PSD experiments, in which sleep homeostatic mitigation occurs by partial daily sleep recuperation of sleep [27], [28]. Because of this and other reported differences in behavioral and physiological responses to chronic PSD and acute TSD [27], [28], it is possible that specific candidate genes play different roles in the degree of vulnerability and/or resilience to the neurobehavioral and homeostatic effects of these two conditions. In further support of this possibility, we recently found that the *PERIOD3* VNTR polymorphism did not relate to individual differences in neurobehavioral performance responses to chronic PSD [24], contrasting data from TSD conditions [29]. Future studies should investigate the distinction between PSD and TSD and the manner in which these conditions relate to phenotype-genotype interactions.

The *Met/Met* homeostatic response to sleep loss may possibly relate to several recent reports of differential responses to treatment in depression as a function of the *COMT* Val158Met genotype. For example, this genotype predicted better antidepressant treatment outcome in major depressive disorder [30]–[32]. Similarly, Benedetti et al. [33] found that bipolar *Met/Met* patients showed better antidepressant response to the combined chronotherapeutic treatments of sleep deprivation and bright light therapy.

Beyond treatment response, we speculate that the faster dissipation of sleep drive in *Met/Met* individuals during exposures to sleep loss may mitigate the development of psychotic features of psychiatric disorders. For example, the *Met/Met* genotype has been related to a reduced risk of experiencing psychotic episodes in bipolar disorder [34] and has been associated with lower severity of delusions in schizophrenia [35]. Whether genotype-related differential responses to sleep loss are important for preventing the development or exacerbation of clinical symptoms is an important area of future investigation in adults with psychiatric disorders.

We found that *Met/Met* individuals had higher IQ scores than *Val/Val* individuals, as has been reported previously in the literature [5], [7]. Even after correcting for this IQ difference, we failed to detect differences across genotypes on a variety of executive functioning or cognitive tasks, in contrast to other reports [1]-[4]. We also failed to detect differences in PVT performance at baseline or during PSD, in agreement with findings from a study in TSD [9], [10]. Similarly, we found no DS performance differences across genotypes, in concurrence with other studies in healthy adults [36], [37]. Moreover, a study in TSD conditions reported no genotype differences in the 2-back test or the random number generation task [8] and a recent large study in healthy subjects also failed to find genotype differences [36]. Our negative results extend meta-analytic results indicating the *COMT* Val158Met polymorphism exerts small effects on executive tasks [7], and support the notion that this polymorphism's role is not generalizable to all cognitive tests or to complex cognitive phenotypes [1], [4], [37].

*Met/Met* subjects showed higher sleep homeostatic pressure during PSD, but not poorer cognitive, executive functioning or subjective sleepiness responses. Such a separation has been noted previously whereby the homeostatic sleep responses to chronic PSD or to TSD have not been reflected in waking neurobehavioral or cognitive responses [9], [10], [24], [27], [38]. We have yet to identify candidate genes that mediate differential vulnerability to cognitive changes resulting from PSD.

All genotypes showed greater physiological sleepiness, sleep homeostasis, and self-rated sleepiness and fatigue, and poorer cognitive performance across PSD. Thus, PSD produced substantial changes characteristic of cumulative sleep loss, thereby validating our phenotypic approach [24], [27], [28], [38]–[40].

Even though we utilized a large sample size compared to all other candidate gene studies investigating response to sleep loss in healthy adults [8]–[10], [29], [41], [42], our findings should be considered preliminary. They serve as a starting point for future—and critical—replication in separate populations.

In addition to the need to replicate our findings, our study has a few limitations. First, we were unable to assess the menstrual cycle phase of our female participants. Second, it is possible that the small but significant genotype difference in REM sleep latency at baseline may affect the relative SWE/SWA values at SR1 and SR5 [43]. Finally, it is possible that genotype differences in NREM-REM sleep cycle lengths [44] may be present during baseline and sleep restriction nights and may influence SWE/SWA hourly values.

In summary, during chronic partial sleep deprivation, *Met/Met* subjects exhibited faster sleep homeostatic dissipation than *Val/Val* subjects. The *COMT* Val158Met polymorphism related to individual differences in sleep homeostatic, but not executive functioning and cognitive responses to chronic PSD, suggesting these measures may be orthogonal and associated with distinct genetic mechanisms. Thus, the *COMT* Val158Met polymorphism may be a biomarker for predicting differential sleep responses resulting from sleep deprivation in healthy adults and by extension, in various psychiatric populations. We speculate that the sharper dissipation of sleep homeostasis in *Met/Met* individuals may be protective against the development of psychosis in bipolar depression and schizophrenia, when exposure to sleep loss occurs, and predictive of antidepressant treatment response—these research areas merit further investigation.
